# A Research on the Sharing Platform of Wild Bird Data in Yunnan Province Based on Blockchain and Interstellar File System

**DOI:** 10.3390/s22186961

**Published:** 2022-09-14

**Authors:** Huaiyuan Yang, Yucheng Li, Hua Zhou, Yili Zhao, Lei Song

**Affiliations:** College of Big Data and Intelligent Engineering, Southwest Forestry University, Kunming 650224, China

**Keywords:** blockchain, bird big data, interplanetary file system, data sharing

## Abstract

Sharing scientific data is an effective means to rationally exploit scientific data and is vital to promote the development of the industrial chain and improve the level of science and technology. In recent years, the popularity of the open data platform has increased, but problems remain, including imperfect system architecture, unsound privacy and security, and non-standardized interaction data. To address these problems, the blockchain’s decentralization, smart contracts, distributed storage, and other features can be used as the core technology for open data systems. This paper addresses the problems of opening, allocation-right confirmation, sharing, and rational use of wild-bird data from Yunnan Province, China. A data storage model is proposed based on the blockchain and interstellar file system and is applied to wild-bird data to overcome the mutual distrust between ornithology institutions in the collaborative processing and data storage of bird data. The model provides secure storage and secure access control of bird data in the cloud, thereby ensuring the decentralized and secure storage of wild-bird data for multiple research institutions.

## 1. Introduction

With the development of artificial intelligence, cloud computing, quantum computing, and blockchain technology, we have entered the era of big data, which is now growing at an unprecedented rate. The results of big-data research are subtly infiltrating all aspects of social economy and daily life and are gradually becoming a growth point of development and innovation. Big data thus plays an increasingly important role in people’s daily life and work [1,2,3]. At present, big data affects all walks of life. For example, the medical field uses doctor–patient data to establish a patient privacy model [4,5]; the financial sector uses market data to analyze market trends [6]. Remote-sensing data are used to analyze the degree of economic prosperity [7], and the agricultural sector uses weather data to predict yields for the coming year [8,9]. The value of data is being increasingly tapped, bringing economic and social benefits.

The exploitation of big data initially lacked authoritative standards and guidance for the processing, measurement standards, and applications of data produced by different scientific institutions. For data on wild birds from Yunnan Province, China, few application programming interfaces (APIs) offer services, which prevents the public and scientific research institutions from optimally mining these data through an open data mining system, thereby curtailing our ability to protect wild birds in Yunnan Province. Thus, the supply of bird-data services lags behind the demand.

Scientific institutions are concerned about security risks, such as data leakage and tampering and user-operation traces [10,11], which is a key factor limiting the sharing of wild-bird data. The cloud data encryption technologies commonly used by most scientific institutions, such as RSA, digital certificate, and the HTTPS protocol, all suffer from data security problems regarding data transmission, data processing, data storage, and authentication sharing in the cloud [12,13,14]. Data are easily intercepted in the network and identity tokens can be forged [15], resulting in asset leakage and the waste of shared platform resources [16,17,18,19]. Various research institutions have open-source sharing platforms for bird data, but these systems mostly have centralized designs. When the cloud service resource center fails, is attacked, or faces other problems, bird data and all big data face serious security risks.

To solve the problems of openness, right allocation, sharing, and the rational use of wild-bird data in Yunnan Province, we propose herein an open platform for bird data based on a blockchain and the InterPlanetary File System (IPFS) [20,21,22,23]. The system stores large files containing bird data in the IPFS in Yunnan Province and uploads to the blockchain the address characters generated by the IPFS, focusing on the management and sharing of the data. A BigchainDB [24], is used to upload the data to the blockchain and to ensure the integrity and reliability of the data. We use smart contracts [25,26,27], and consensus mechanisms [28,29], to provide full-process shared security services. The proposed system allows the open management and rational use of bird data from Yunnan Province, which is vital for ornithology.

This paper will describe in detail the blockchain and interstellar file system-based data sharing platform for wild birds in Yunnan Province from ‘Related preparatory work’; ‘Platform architecture and module design’; ‘Experiment’; ‘Experimental results and analysis’ and ‘Conclusions’.

## 2. Related Work

### 2.1. Smart Contract

Smart contracts were proposed by cryptographer Szabo in 1994. These involve a transaction protocol that can be deployed in a cloud service network. Smart contracts provide the specific functions of the blockchain network. Because smart contracts are based on blockchain technology, digital signature-based contracts are verifiable, immutable, and irrevocable contracts [30,31]. Typically, smart contracts are authenticated by network nodes and run as code on the blockchain [32,33]. Once the preset conditions of the smart contract are met, the smart contract automatically executes and is not affected by the outside world.

The smart-contract layer encapsulates various types of script codes, algorithms, and complex smart contracts, so it is the basis for flexible programming and the operation of blockchain systems. The application of blockchain smart contracts in the process of accessing bird data not only promotes the access of bird data but also improves the standardization of multi-dimensional uplink, data authentication, data opening, data application, and traceability of bird data.

### 2.2. Consensus Mechanism

In the peer-to-peer network model, network communication problems lead to modifications in the order in which each node receives transactions. The role of the consensus mechanism is to synchronize the information obtained by all nodes in the network, thereby ensuring the consistency of each node in the blockchain network [34]. Different application scenarios have their appropriate consensus mechanisms. The current mainstream blockchain consensus algorithms [35], include PoW, PoS [29,36], practical Byzantine fault tolerance (PBFT) [37], and Raft [38,39]. PoW has a high safety factor but requires excessive computing resources, and PoS is an efficient consensus mechanism, which is light on computing resources, but has poor security. The PBFT consensus algorithm was proposed by Castro and Liskov and was first used to solve the Byzantine general’s problem. It has (*n* − 1)/3 fault tolerances in a blockchain network composed of a limited number *n* of nodes and ensures a certain level of performance.

### 2.3. InterPlanetary File System

The InterPlanetary file systems (IPFS) can be used for protocols and peer-to-peer networks that store and share multiscale data in distributed file systems in the cloud [21]. IPFS uses content addressing to uniquely identify each file in a global namespace that connects all computing devices, intending to be a standard common file system for all cloud computing devices. The IPFS incorporates the ideas of systems and peer-to-peer networking protocols, including but not limited to distributed hash tables [40], BitTorrent, Git, and SFS [41]. The IPFS can be regarded as an independent bit torrent cluster for object exchange in a Git warehouse. In other words, the IPFS provides a high-throughput content-based block storage model with content hyperlinks [42]. In a multi-level hierarchical storage system, each storage system has its data structure, and each data structure is its database. This creates an extensive Merkle DAG data structure that can be used to build various versions of file systems, blockchains, and even permanent websites. The IPFS combines distributed hash tables and block exchange with an incentive mechanism and a self-certified namespace. The IPFS has no single point of failure, and nodes do not need to trust each other.

In terms of data sharing and storage, the IPFS can integrate the same files, reducing data redundancy and thereby economizing the storage control of cloud resources. After the data are uploaded to the IPFS for a hash calculation, the system generates a hash value corresponding to the unique file format, such as 5cee88f94a94df73199f805f6df51bb3d6ab6e540494494c78bf7ed0d6f52fa23dc91a8c62e5a272ee43cffe182a25b0d799c392ab6d7e1b3efba1cfc5d60836. The generated hash value is uniquely associated with the information content of the stored data. When the data are even slightly updated, a completely different hash value is produced. When a file is sought, the IPFS combines and removes the file from the distributed storage node according to the previously generated file fingerprint, verifies the file, and returns it to the requesting user. At the same time, the IPFS promotes positive and negative feedback from each node in the file storage system while accounting for the storage system structure and respecting the universal law of files.

### 2.4. BigchainDB

A blockchain is a distributed database or public ledger and has features, such as decentralization, trustlessness, high transparency, and traceability. The BigchainDB is a database with high throughput, low latency, powerful query capabilities, decentralized control, immutable data storage, and built-in asset support, just like a blockchain.

BigchainDB 2.0 [43], uses Tendermint for all networks and consensus. Each node has its local database, and all communication between nodes is conducted by using the Tendermint protocol. Tendermint is a secure state machine replication algorithm in the blockchain paradigm. Its algorithm form is BFT-ABC and has an additional accountability system to facilitate the verification of dishonest behavior of Byzantine nodes. If any node fails, the rest of the network continues to function. The following features of BigchainDB guarantee the needs of different users:

(1) User data are stored on the BigchainDB network and cannot be altered or erased.
(2) The concept of the BigchainDB owner controlling an asset implies that only the owner of the asset can transfer the asset.
(3) The ability to process large numbers of transactions per second has always been one of the design goals of BigchainDB, and the BigchainDB network can include transactions in newly committed blocks in just a few seconds.
(4) Each node in the BigchainDB 2.0 network has its own local MongoDB database. Every node operator can use the full power of MongoDB to index and query stored data.

## 3. Platform Architecture and Module Design

### 3.1. Platform Architecture

**Figure 1:**
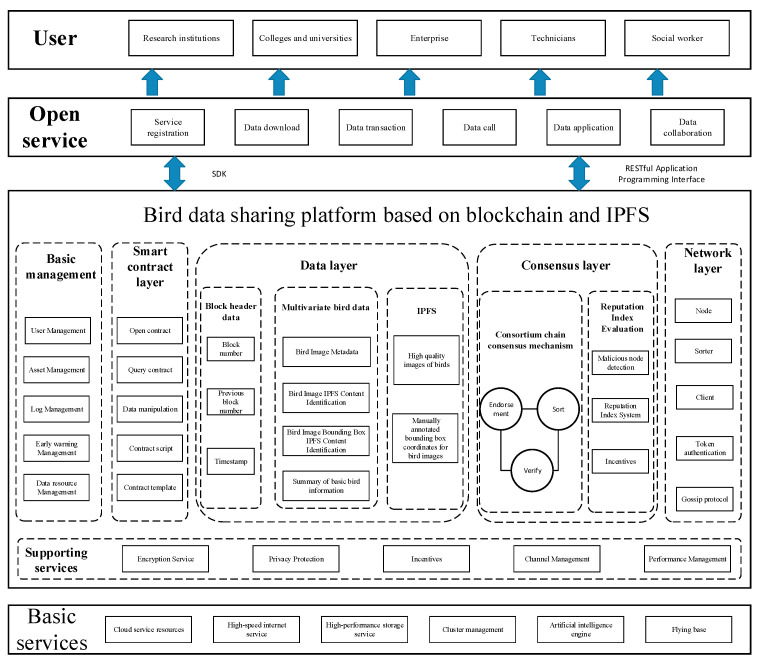
Blockchain-based open platform architecture for bird data.

Given the current problems, such as data development and data risks, we propose herein a blockchain-based open-platform architecture that we test on bird data (see Figure 1). The architecture is divided into basic services, supporting services, basic management, smart contract layer, data layer, consensus layer, network layer, user layer, and open services to establish a set of open platforms for bird data that can satisfy multiple user categories, with complete functions and efficient performance, and ensure the source and ownership of data. In this scheme, blockchain technology is mainly used for the smart contract layer and data storage layer.

1. User module

For user groups, the system is mainly divided into scientific research institutions, universities, enterprises, scientific researchers, and social workers, and each user group establishes different identities and divides the ownership accordingly to ensure the integrity of the platform architecture.

2. Open Services

The system provides open services, such as user service registration, data download, data transaction, data call, data application, and data collaboration. Users can request relevant services on the platform, and the platform can generate interface feedback for users. The release service module, which mainly performs the open service for upper-level users of bird data, is the business hub of the bird data open service. This module provides a unified API and software development kit (SDK) in mainstream development languages for the blockchain-based open platform for bird data. Upper-level users can quickly access the platform’s service registration, data download, data transaction, data call, data application, and data collaboration services through the API and SDK.

3. Blockchain-based open platform for bird data

The open platform for bird data contains six modules: basic management, smart contract layer, data layer, consensus layer, network layer, and support services. It mainly provides comprehensive and integrated services for the basic operation and management of the open bird-data platform, including user management, asset management, log management, early warning management, and bird-data resource management.

### 3.2. Platform Module Design

#### 3.2.1. Basic Management Module

The basic management module is mainly for the management of platform users, assets, logs, security alerts, data resources, etc., to guarantee the operation and maintenance of the whole system.

#### 3.2.2. Open Services

The smart contract layer is mainly written based on the business contracts of each node or participant, defining a series of transaction processing processes, including business contracts, query contracts, data call modules, asset turnover modules, and contract templates. The business contract is the concrete embodiment of the business rules for platform operation, which is the basis of platform operation. The query contract is convenient for external invocation of the platform smart contract and authentication of the platform smart contract. The data invocation module adds, retrieves, and performs other operations on the data in the blockchain.

#### 3.2.3. Data Layer

The main role of the data storage layer is to carry out regular storage and on-chain distributed storage of platform data. The storage methods in the data storage layer can be divided into two types: cloud storage and the BigChainDB-based blockchain system. Cloud storage mainly serves conventional static resources or basic business resources. Commonly used cloud storage applications include the Remote Dictionary Service, the Object Storage Service, and TencentDB. The blockchain system based on BigChainDB supports data block formation, asymmetric cryptographic validation, a decentralized ecosystem, a consensus mechanism, and distributed storage to ensure the security and traceability of business data.

#### 3.2.4. Consensus Layer

We use the Byzantine fault-tolerance mechanism as the consensus layer consensus algorithm, which enables each node on the blockchain or each coalition member to establish a coalition network whose nodes can trust each other so that each node in the entire coalition network can complete transactions quickly and accurately. This ensures that the data stored in each node can receive millisecond-level real-time synchronous updates and multi-node data consistency, to realize the safety and reliability of asset information and log records in the blockchain-based system.

#### 3.2.5. Network Layer

The network layer mainly provides Internet data interaction and transmission services for the open platform of bird data, including endorsement nodes, sorters, bookkeeping nodes, token authentication, and the configuration of the Gossip protocol.

#### 3.2.6. Matching Services

Based on the platform that provides the corresponding encryption, privacy protection, incentive mechanism, channel management, performance management, and other supporting services, the model provides the corresponding resource services while protecting the platform number, which promotes the use of the platform.

This study thus designs and implements a blockchain-based open platform for bird data based on the above system architecture and module design combined with a blockchain data management system. The integration of multi-dimensional data on birds provides an important service to governments, ornithologists, and research institutions, and facilitates research into biodiversity and conservation.

### 3.3. Blockchain- and IPFS-Based Data Storage Model for Birds

For this paper, we add 100 species of birds with multidimensional data, ten dimensions of bird information, and the accompanying feature data to YUB-300-2019 [44], and name the dataset “YUB-400-2021.” Many high-quality image files of Yunnan wild birds and manually labeled bracketed box files form the core and value of the YUB-400-2021 dataset, and the blockchain is extremely expensive for storing large files. Uploading the image files of Yunnan wild birds along with manually annotated enclosed-box JSON files as data causes two problems:

1. Large files occupy a large amount of bandwidth during network transmission, which degrades somewhat the input-output performance of the blockchain network.
2. Hypertext data require more time for data unchaining, which reduces the efficiency of data up-chaining and consumes extra time when physical users or scientific institutions pull the complete data into the blockchain.

**Figure 2:**
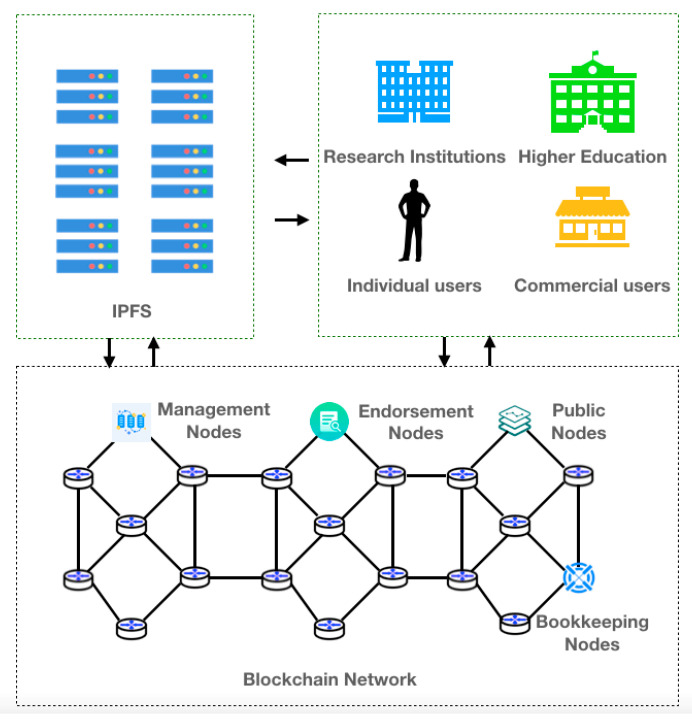
Blockchain- and IPFS-based data storage model for birds.

**Figure 3:**
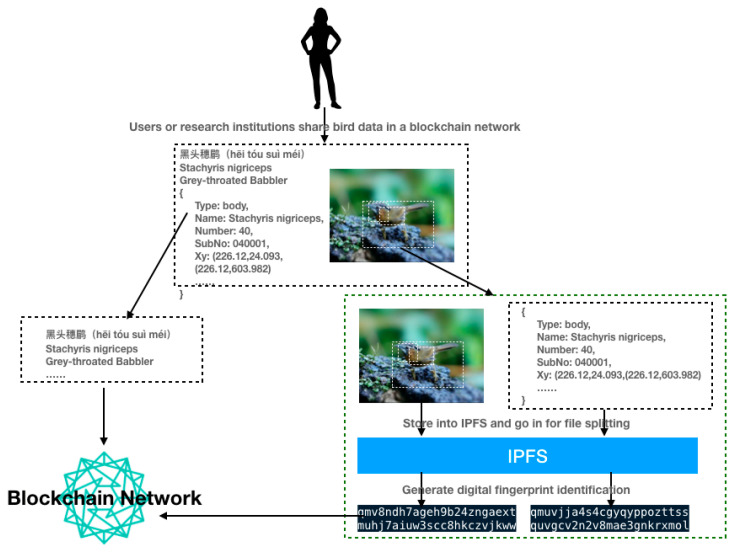
Schematic diagram of Stachyris nigriceps data uploading to the blockchain.

To solve these problems, we introduce the IPFS to deal with the problem of storing large data files and propose a bird-data-sharing model based on a blockchain and the IPFS. Figure 2 shows the blockchain- and IPFS-based data storage model for birds. In this mode, we further divide the YUB-400-2021 dataset into base data and file data, and the user or research institution stores the file data in the IPFS. When a user or research institution adds a file to the IPFS, the uploaded file is split into small chunks, cryptographically hashed, and given a point-in-time-based digital fingerprint identifier called the content identity (CID), which is used as a permanent record for this file. The uplinked data are the CID corresponding to the bird base data and the base data file. Consider Stachyris nigriceps in YUB-400-2021 as an example, as shown in Figure 3. Bird data storage pattern based on blockchain and IPFS characteristics table as shown in Table 1.

Users or research institutions share bird data in a blockchain network. The data are split into general data, such as the Chinese name “Black-Headed Thrush,” the English name “Stachyris nigriceps,” and related base data in the figure, and large file data, such as the Black-Headed Thrush image, and the enclosing box coordinates JSON file. The large file data are stored in the IPFS, which slices the large files and generates the digital fingerprint identification. The Stachyris nigriceps large file digital fingerprint identification is stored in the blockchain along with the general data combination.

### 3.4. Blockchain- and IPFS-Based Data-Sharing Model for Birds

**Figure 4:**
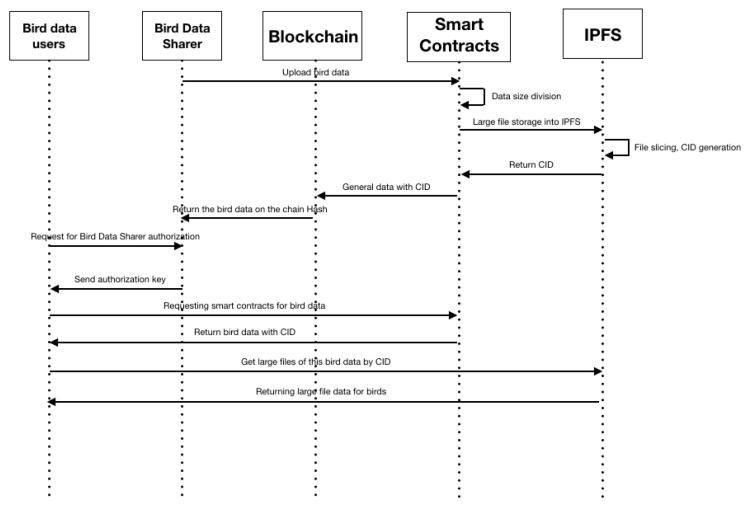
Blockchain and IPFS-based data sharing model for birds.

The blockchain- and IPFS-based bird data-sharing model has the features of blockchain decentralization and tamper-proof and reviewable IPFS distribution, which differs significantly from the centralized and integrated model used in traditional bird-data sharing. The blockchain- and IPFS-based bird data-sharing model is shown in Figure 4. Five subjects are included in the sharing process: bird data sharer, bird data user, blockchain, smart contract, and IPFS.

The bird data sharers are the most central aspect of this sharing model. Bird data sharers are the uploaders, authenticators, and licensors of bird data. We purchased seven Elastic Compute instances on AliCloud at a unit price of US $3200/year for the deployment of our storage services. Five of them are mounted with the blockchain database BigchainDB and the other two are mounted with Go-IPFS v0.10.0. The blockchain databases BigchainDB and IPFS are both free open-source software that does not need to be paid for. Because the platform does not involve users in monetary transactions, there are also no costs to users in terms of monetary transactions.

### 3.5. Web Interface Service Design

The web interface mainly serves to collaborate with external applications, generally with command script calls and high-level programming language program calls. BigchainDB and IPFS both provide several excellent HTTP client-side service APIs, but the use of The way and reference documents are complicated. When bird data sharing needs to change or the underlying blockchain and file storage forms need to be iterated, all programs in each smart contract need to be modified, which adds a great burden to the second upgrade of the bird sharing platform and daily operation and maintenance. Therefore, the need to design a network interface between BigchainDB and IPFS is very urgent.

Unlike SOAP-based first and second generation Web services, REST is an architectural style designed for business resource objects that is simpler and closer to the original intent of the Web. It is simpler and closer to the original intent of the Web. It frees users from complex and cumbersome business logic to focus more on the development of critical functionality or application components, and can effectively reduce development costs, while also providing good scalability. RESTful uses an architecture designed for business resource objects, so the first thing the RESTful API focuses on at the beginning of its design is available resources. Therefore, all actionable elements present in the business back-end runtime service can be considered as resources, either specific or abstract. By giving full play to the advantages and design principles of RESTful, the business can be separated from the front and back ends at the beginning of the design, and the front-end and back-end data interaction in the form of an interface without worrying about other effects caused by the upgrade of the module, from the perspective of the system architecture, RESTful can effectively improve the compatibility of the overall system. At the same time, taking into account the blockchain and IPFS tamper-evident property, the web interface selects GET and POST requests to the API for business data transmission and reality. To ensure the security of network data transmission, an HTTPS protocol is used.

The API interfaces of the blockchain and IPFS-based bird data sharing platform are listed in Table 2 Core API List.

To ensure the data specification during API usage, the sharing platform will specify a consistent request format for each API. The specific format is shown in Table 3. Detailed API documentation for the system can be found in Supplementary Materials.

### 3.6. User Interface

**Figure 5:**
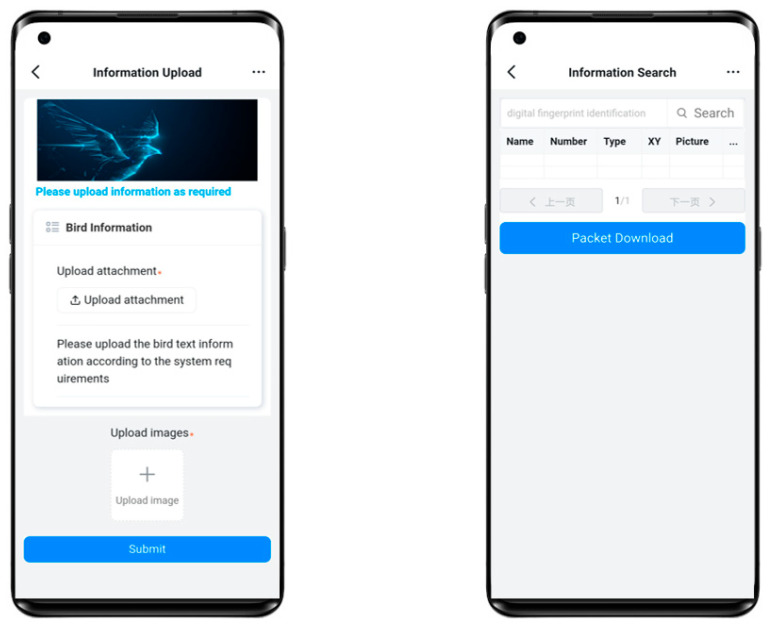
User upload and download interface.

In terms of the use of the system, the system provides an information upload interface and information download interface for users or registered users of research institutions. Because the system is to store the text information of the data in the blockchain first, the large file images are stored in the IPFS to generate the digital fingerprint identification, and then the generated digital fingerprint identification is stored in the blockchain. So users need to divide their collected data sets into text information and large files of images and then upload them to the system. In the data search interface. Users only need to provide the corresponding digital fingerprint identification to search for the required data and can download it. User upload and download interface figure as shown in Figure 5.

## 4. Experiment

Experimentation allowed us to verify that the blockchain- and IPFS-based bird-data-sharing model (Pattern IV) proposed herein outperforms the traditional cloud storage model. We compare sample instance patterns (Pattern I), third-party authorization authentication patterns (Pattern II), and blockchain-based bird-data-storage patterns (Pattern III).

### 4.1. Sample Instance Pattern

**Figure 6:**
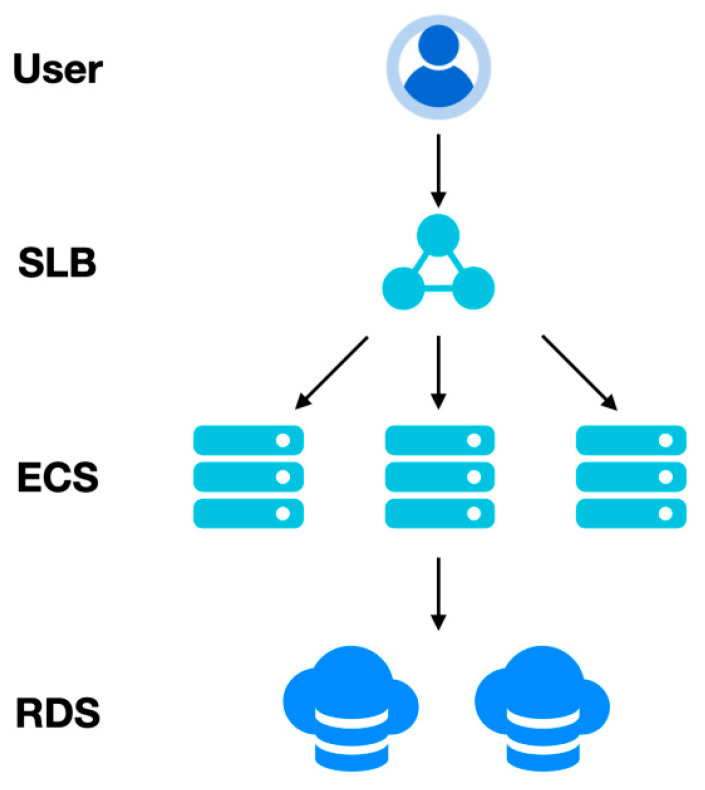
Sample instance pattern.

The sample instance pattern is a widely accepted data storage pattern. The composition of this model is relatively simple, and the core components are the elastic compute service and the relational database service, where the former is mainly used for logical computation and data flow, and the latter is mainly used for data storage and backup. The single instance pattern is combined with server load balancing as an external service. Sample instance pattern figure as shown in Figure 6. Single-instance pattern characteristics table as shown in Table 4.

### 4.2. Third-Party Authorization Authentication Pattern

**Figure 7:**
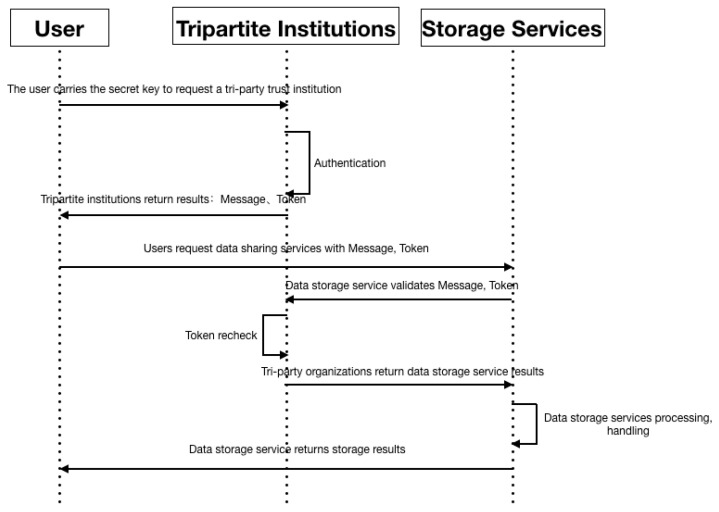
Third-party authorization authentication pattern.

The third-party authorization authentication pattern (Pattern II) is created in the data storage process that developed during the explosive growth of data demand and user volume. Three-party authorization authentication is conducted by the authentication layer at the core of the model. During data sharing, the third-party certification authority is trusted unconditionally. All validation operations are performed by the third-party certification authority and the results are returned. The authentication methods of third-party organizations include but are not limited to token timing, secret key verification, symmetric encryption, and attribute encryption. The third-party authorization authentication pattern figure is shown in Figure 7. Third-party authorization authentication pattern characteristics table as shown in Table 5.

In the three-party authorization authentication mode, the steps to complete for data storage are as follows:

- The user requests the third-party trust authority with its secret key.
- The third-party authority confirms the authentication.
- The third-party organization returns the result message and token to the user.
- The user uses the message and token to request the data storage service.
- The data storage service requests the third-party institution to verify the message and token.
- The third-party trust authority revalidates the token.
- After verification, the third-party trust agency returns the result to the data-storage service.
- The data-storage service processes and handles the data.
- The data-storage service returns the storage result.

### 4.3. Blockchain-Based Bird-Data-Storage Pattern

**Figure 8:**
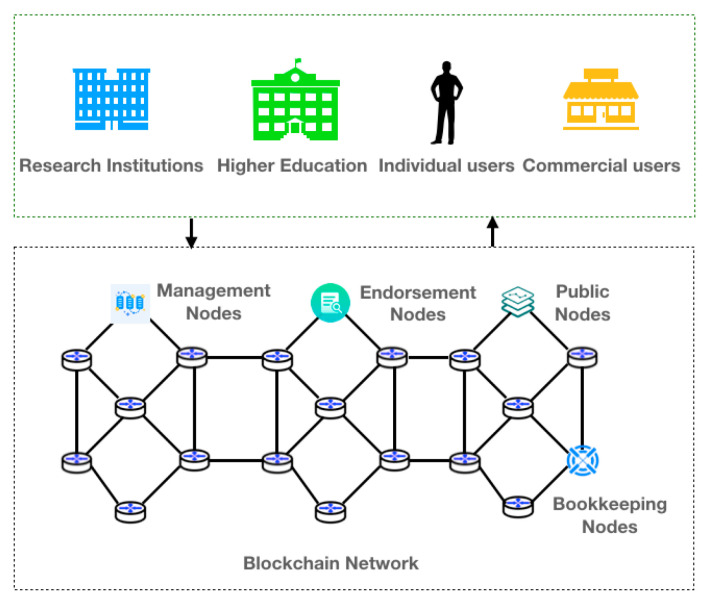
Blockchain-based data storage model for birds.

The blockchain-based bird data storage pattern categorizes user groups into research institutions, universities, individual users, and commercial users, and the blockchain network contains management nodes, endorsement nodes, public nodes, and bookkeeping nodes. Figure 8 shows the blockchain-based data-storage model for birds.

**Figure 9:**
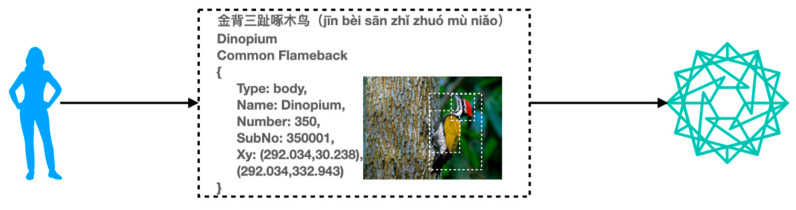
Schematic diagram of Dinopium data uplink based on Pattern III.

Based on Pattern III, users or scientific institutions can directly package and upload all bird data to the blockchain. The bird data are exemplified by the Dinopium Javanese in the YUB-400-2021 dataset (see Figure 9). Blockchain-based bird data storage pattern characteristics table as shown in Table 6.

### 4.4. Service Resource

For the three models proposed above and the proposed bird-data-storage pattern based on the blockchain and IPFS (Pattern IV), four groups of cloud service resources are constructed in the data pool.

Pattern I has four elastic computing instances, one of which is configured with four cores of 8 GB of memory and 50 GB storage for server load balancing. The remaining three are configured with eight cores with 16 GB of memory and 200 GB storage for business computing, We have two relational database instances, both configured with two-core 4 GB capacity and 500 GB storage for storing business data. As shown in Table 7.

For Pattern II, the storage service is the same as for Pattern I: we have four additional elastic compute instances, one of which is configured with two cores and 4 GB of memory, and 100 GB of storage for server load balancing. The remaining three are configured with eight cores and 16 GB of memory for business computing services. The operating system is Linux 7.2. The remaining three are configured with eight cores and 16 GB of memory, 500 GB of storage for business computing services, and Linux 7.2 as the operating system. There are two relational database instances, and the configuration is two cores with 4 GB of memory and 500 GB of storage for storing business data. As shown in Table 8.

Pattern III has five resilient computing instances, all configured with eight cores, 16 GB of memory, 500 GB of storage, and the Ubuntu 18.04 operating system. As shown in Table 9.

In Pattern IV, on top of Pattern III, two additional elastic computing instances are added and are configured with eight-core 16 GB of memory and 500 GB of storage, mounted with the IPFS of Go-IPFS v0.10.0, and the BigchainDB service is also deployed. Node storage can be reduced to 200 GB. As shown in Table 10.

## 5. Experimental Results and Analysis

**Figure 10:**
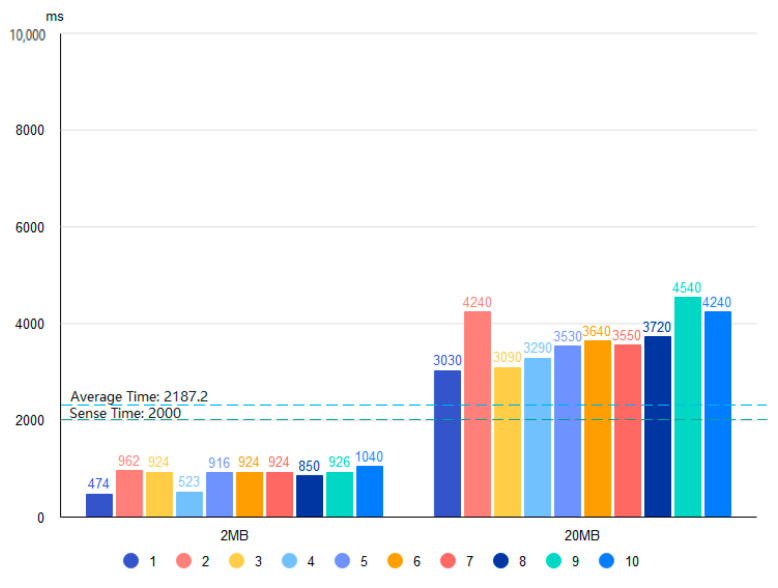
Time test of Pattern I.

**Figure 11:**
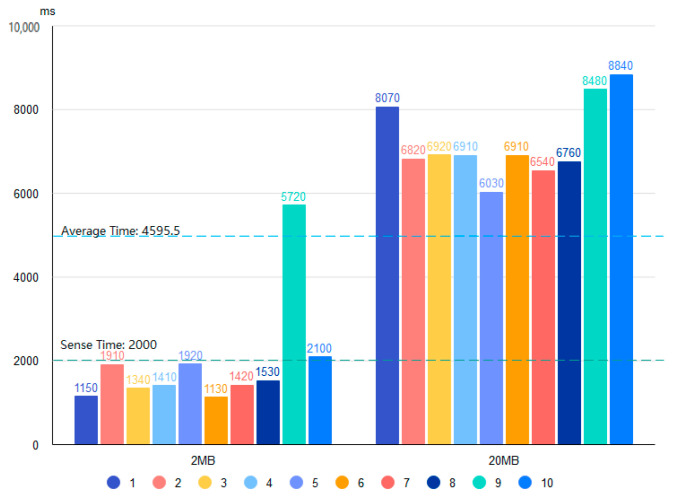
Time test of Pattern II.

**Figure 12:**
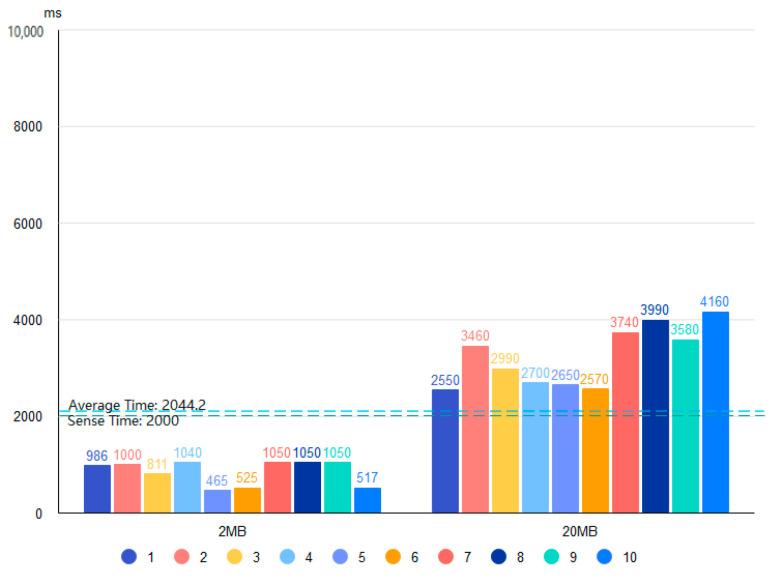
Time test of Pattern III.

**Figure 13:**
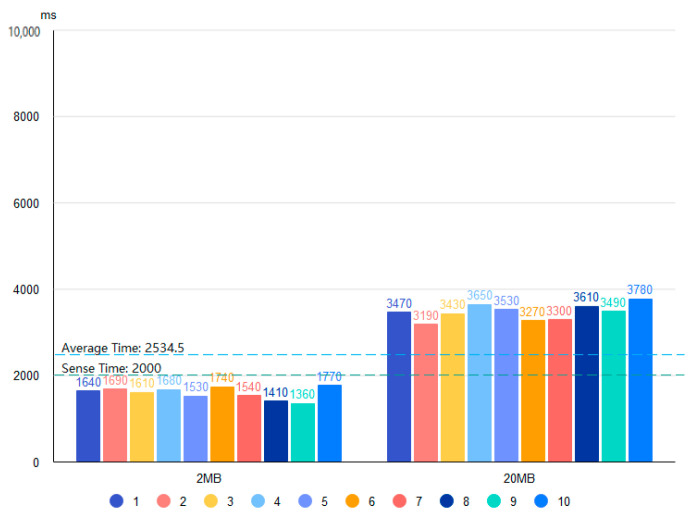
Time test of Pattern IV.

Based on the four proposed data storage modes and the deployment environment, we use 2 and 20 MB files containing bird data and conduct 20 tests for each of the four storage systems. Figure 10, Figure 11, Figure 12 and Figure 13 show the results of the Pattern I–IV time-consumption test, respectively. The results are grouped by file size; the abscissa is the time (ms), the ordinate is the time required to store (ms), the blue dashed line marks the overall average time taken in this mode (ms), and the green dashed line is the upper limit of user senseless delay (ms).

**Figure 14:**
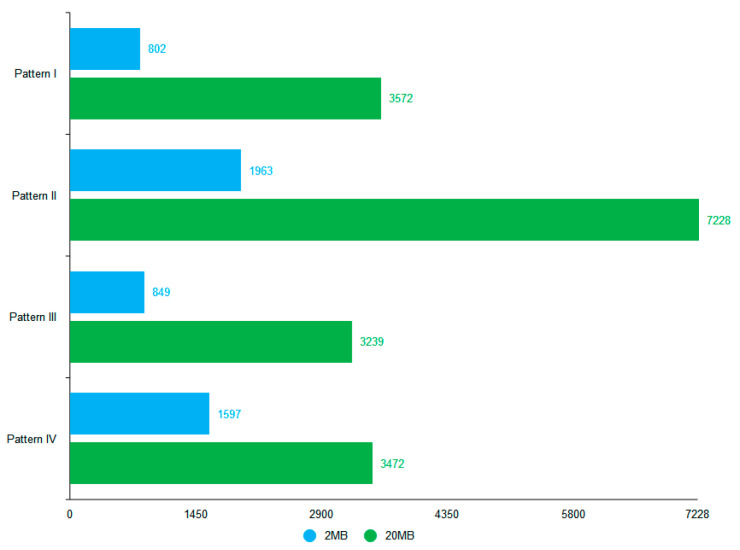
Histogram of time consumption of data storage in the four patterns with different file sizes.

Combined with Figure 14, the average time consumption of the four patterns for storing data in different file sizes is shown in the histogram. Figure 10 shows the Pattern I time consumption test, which gives the lowest average storage time (802.4 ms) of the four modes for Pattern I with a file size of 2 MB. The overall average storage time is close to the upper limit of user insensitive latency among the four modes, which is in line with the general user experience.

Figure 11 shows the Pattern II time consumption test, which produces the largest average storage time (7228 s) of the four modes for a file size of 20 MB. Of the four modes, the overall average storage time is the farthest from the user-perceived delay time, which is due to the time-consuming practice whereby data are passed back and forth between three parties. In addition, too many communications take place between the ends, which causes fluctuations in time consumption.

Figure 12 shows the Pattern III time consumption test. Pattern III is a better pattern with decentralized and tamper-proof blockchain features, such as the average time consumption of Pattern I in the cases of 2 and 20 MB file sizes. The time consumption test of Pattern IV in Figure 13 shows that the average storage time for Pattern IV is about twice that of Pattern III when the file size is 2 MB. This is because the bird data are transferred by first storing the file to the IPFS and then uploading the address of the IPFS to the blockchain. The distributed storage nature of IPFS can also affect the transfer times. However, the average storage time of Pattern IV with a 20 MB file size is like that of Patterns I and III but is more stable. It ensures good performance in situations where large files occupy significant bandwidth during network transmission, which affects the input-output performance of the blockchain network. Note that hypertext data will take longer to transfer to the blockchain.

Given a 2 MB (20 MB) bird image file, the average time for Pattern I (III) is chosen as the reference.

Table 11 shows that the patterns ranked from fast to slow are Pattern I, Pattern III, Pattern IV, and Pattern II in the horizontal case based on Pattern I with a file size of 2 MB. Table 12 shows that the patterns ranked from fast to slow are Pattern III, Pattern IV, Pattern I, and Pattern II in the horizontal case based on Pattern III with a file size of 20 MB. In summary, Pattern IV is the best solution to ensure the security of the bird data, efficient transmission, and cost-effectiveness.

## 6. Conclusions

This paper compares the system structure, modules, and operation mechanism under various data storage modes for data on the diverse wild birds of Yunnan Province. A multi-domain complex system is formed by organically combining the idea of blockchain multi-node decentralization with IPFS distributed slicing storage. The paper describes in detail the evolution of blockchain-based sharing data on the wild birds of Yunnan Province.

The main contribution of this paper is to extend the wild bird dataset YUB-200-2017 of Yunnan Province (named YUB-400-2021), which now has 400 bird species and 24,000 bird images involving 15 orders, 57 families, and 178 genera. Second, a blockchain and interstellar file system-based storage model for wild bird data in Yunnan Province is proposed to solve the problem of mutual distrust between bird research institutions in collaborative bird data processing and data storage. The existing models are analyzed in detail and the advantages and shortcomings of each are discussed. Based on blockchain technology, we use the interPlanetary File System technology to store large files of bird data and use blockchain encryption technology to ensure secure storage and secure access authority control in the cloud. This approach decentralizes and secures the storage of data from ornithology institutions. Finally, we design a platform for sharing data on wild birds in Yunnan Province based on a blockchain and Interplanetary File System. We describe in detail the architecture and modular design of the blockchain- and IPFS-based bird-data-sharing platform. We also highlight the main smart contracts and consensus mechanism design concepts and the operational mechanism of the sharing model involved in the bird-data-sharing platform. Finally, the design and construction of the platform’s external web service interface are described in detail.

There are still many unfinished aspects of blockchain and IPFS-based research on wild bird data sharing in Yunnan that need further study. In this paper, the PBFT consensus algorithm is selected for the bird data sharing platform based on blockchain and IPFS, but the PBFT consensus algorithm also has shortcomings. Therefore, in the next step, we need to further optimize the design of the platform consensus algorithm and improve the platform performance.

## Figures and Tables

**Table 1:** Bird data storage pattern based on blockchain and IPFS characteristics table.

Pattern Name	Benefits	Inadequate
Bird data storage pattern based on blockchain and IPFS	1: Decentralized. Each node in the network is independent and equal in the blockchain network 2: Secure and reliable. The blockchain is deployed in a distributed manner, with each node in the network having data on the blockchain 3: IPFS can integrate the same files in data sharing and storage, reducing the redundancy of file data, thus saving the storage control of cloud service resources	1: Large files occupy a large amount of bandwidth during network transmission 2: Hypertext data require more time for data to unchain

**Table 2:** Core API List.

Interface Name	Type	Function Description
/open/ipfs/v1/getDocumentByAddress	GET	Search documents by address
/open/ipfs/v1/insertDocument	POST	Storage files
/open/ipfs/v1/updateDocument	POST	Update files
/open/bigchaindb/v1/getIndividualDetail	GET	Obtain information about users on the blockchain
/open/bigchaindb/v1/getPeerStatus	GET	Query Node Status
/open/bigchaindb/v1/registerIndividualAccount	POST	Register Account
/open/bigchaindb/v1/configChainProperties	POST	Configure blockchain property files

**Table 3:** API request body format.

Response Code	Response Message	Processing Method
0	Success	Response successful
400001	TS_AUTH_ERROR	Authentication error
400002	TS_ENCRYPTION_EXCEPTION	Decryption exceptions
400003	TS_PEER_EXCEPTION	Node Anomaly
……	……	……
500001	TS_REQUEST_FREQUENCY_LIMITED	Request frequency is limited, please try later
500002	TS_ERROR_PERMISSION	Insufficient Permissions

**Table 4:** Single-instance pattern characteristics table.

Pattern Name	Benefits	Inadequate
Sample Instance Pattern	1: Basic data storage needs are met 2: Support data transmission to any node of the network via the Internet 3: Data are fully encrypted during data transmission	1: No mandatory SSL certificate for cloud-based open applications 2: There is a risk of a data breach

**Table 5:** Third-party authorization authentication pattern characteristics table.

Pattern Name	Benefits	Inadequate
Third-party authorization authentication pattern	1: Basic data storage needs are met 2: There will be a mandatory token bucket policy under high-frequency access	1: The centralization problem of third-party trust institutions is prominent 2: There will be a mandatory token bucket policy under high-frequency access

**Table 6:** Blockchain-based bird data storage pattern characteristics table.

Pattern Name	Benefits	Inadequate
Blockchain-based bird data storage pattern	1: Decentralized. Each node in the network is independent and equal in the blockchain network 2: Secure and reliable. The blockchain is deployed in a distributed manner, with each node in the network having data on the blockchain	1: Large files will occupy a lot of bandwidth during network transmission, which will affect the performance of the blockchain network

**Table 7:** Cloud resource list for Pattern I.

Service Name	Operating System	Quantity	vCPU	Memory (GB)	Storage (GB)
Elastic Computing Instances	Linux 7.2	1	4	8	50
Elastic Computing Instances	Linux 7.2	3	8	16	200
Relational Database Instances	Linux 7.2	2	2	4	500

**Table 8:** Cloud resource list for Pattern II.

Service Name	Operating System	Quantity	vCPU	Memory (GB)	Storage (GB)
Elastic Computing Instances	Linux 7.2	1	4	8	50
Elastic Computing Instances	Linux 7.2	1	2	4	100
Elastic Computing Instances	Linux 7.2	6	8	16	200
Relational Database Instances	Linux 7.2	4	2	4	500

**Table 9:** Cloud resource list for Pattern III.

Service Name	Operating System	Quantity	vCPU	Memory (GB)	Storage (GB)
Elastic Computing Instances	Ubuntu 18.04	5	8	16	500

**Table 10:** Cloud resource list for Pattern IV.

Service Name	Operating System	Quantity	vCPU	Memory (GB)	Storage (GB)
Elastic Computing Instances	Ubuntu 18.04	7	8	16	500

**Table 11:** Cross-sectional ranking based on Pattern I for a 2 MB file.

Model Name	Comparison with Pattern I	Rank
Pattern I	100%	1
Pattern III	105.86%	2
Pattern IV	199.03%	3
Pattern II	242.82%	4

**Table 12:** Cross-sectional comparison based on Pattern I for a 20 MB file size.

Model Name	Comparison with Pattern III	Rank
Pattern III	100%	1
Pattern IV	107.19%	2
Pattern I	110.28%	3
Pattern II	223.16%	4

## Supplementary Materials

The following supporting information can be downloaded at: https://www.mdpi.com/article/10.3390/s22186961/s1, Interface List (Interface documentation for an open platform for bird data based on a blockchain and the IPFS).
