# Supplementary material for: A Research on the Sharing Platform of Wild Bird Data in Yunnan Province Based on Blockchain and Interstellar File System
Source: Sensors (Basel). 2022 Sep 14;22(18):6961. doi: 10.3390/s22186961 (PMC9501809; doi:10.3390/s22186961)
Supplement: Supplementary file 1 [file sensors-22-06961-s001.zip › sensors-1872836-supplementary.pdf]

Interface documentation for an open platform for bird data based on a blockchain and the IPFS

1 Document Introduction......3

1.1 Special Announcement......3

1.2 Reading Object......3

1.3 Product description......3

1.4 Explanation of term......4

1.5 nterface tool test......4

2 Usage process......4

3 Interface List......5

3.1.1 Search documents by address......5

3.1.2 Storage files......7

3.1.3 Update files......9

3.1.4 Get information about users on the blockchain......11

3.1.5 Query Node Status......13

3.1.6 Register Account......15

3.1.7 Configure blockchain property files......17

1 Document Introduction

1.1 Special Announcement

No part of this document may be reproduced or transmitted for any purpose, in any form or by any means, including, but not limited to, mechanical or electronic, without the written permission of a member of the project team. The members of the project team own patents (or have patent applications pending), trademarks, copyrights, or other intellectual property rights in the technology and products covered by this document. This document does not grant a license to these patents, trademarks, copyrights, or other intellectual property rights unless a written license agreement is obtained from a member of the project team.

Any names, company names, and data used in this document for product functionality examples and descriptions are fictitious and are for internal testing purposes only by the project team.

1.2 Reading Object

Development, maintenance, and management staff in the technical department should have a basic knowledge of

1. An understanding of HTTPS/HTTP protocols etc.
2. Understand the basic concepts of information security.
3. Knowledge of at least one programming language for computers.

1.3 Product description

This development manual provides a detailed description of the system's functional interfaces. This guide will provide a comprehensive understanding of the system and enable technical staff to master the system's interfaces as quickly as possible and to be able to develop the system.

1.4 Explanation of term

Abbreviations of nouns	Definition of the noun
Client side	The client in this document is the ApiPost client.
Server-side	The server side in this document is the user side.
Environment variables	Dynamic KV parameters in this system.

1.5 nterface tool test

1. Test tools recommended ApiPost interface test tool. Official address: https://www.apipost.cn

2. The ApiPost test tool is used as follows.

①. Enter the gateway address or interface address into the url correctly.

②. Fill in all the parameters in the order from top to bottom according to the following directory and interface definition.

③. Write the global parameters of the directory or script correctly.

④. Fill in the parameters of the interface and the content of the custom script in the corresponding positions.

3. The custom script supports data collocation with request message encryption.

2 Usage process

1. Preparation phase.

A. Requesting information such as test numbers.

B. Obtaining information such as a development manual (this document).

2. Development phase.

A. quickly become familiar with the docking interface according to the DEMO provided in conjunction with the development documentation.

B. Develop their system according to the interfaces provided by this system to achieve the required business functions.

C. Conduct comprehensive testing of the business functions of their system.

D. Intermodulation with the test environment.

3. Production use.

A. Use the official information provided by the system.

3 Interface List

3.1.1 Search documents by address

Interface Status: Completed

Interface URL：http://domain/open/ipfs/v1/getDocumentByAddress

Content-Type：application/json

Request Method：get

Header parameters and description

Parameter Name	Example values	Parameter Type	Required or not	Parameter Description
accessToken	OfepC2Atn9ZWEgU2bqbJ6rFqDtTrGv2V	String	Yes	
expiredTime	7200	Number	Yes	

Field Description

Parameter Name	Example values	Parameter Type	Required or not	Parameter Description
ipfs_id	EvqKdgbm0esJ1sMB	String	Yes	ipfs_id
address_id	h5witQJeo6IQwz1qU6DNufUrBGmGW21u	String	Yes	address_id
space_id	F7Uo3dbKyUUp8f3	String	Yes	space_id

	EPU45hNvVsTFQwKjC			
doucument_type	detail	String	Yes	doucument_type
doucument_temp_url_address	false	Boolean	Yes	doucument_temp_url_address

Body parameters and description


```
{
    "ipfs_id" :"EvqKdgbm0esJ1sMB",
    "address_id" :"h5witQJeo6IQwz1qU6DNufUrBGmGW21u",
    "space_id" :"F7Uo3dbKyUUp8f3EPU45hNvVsTFQwKjC",
    "doucument_type" :"detail",
    "doucument_temp_url_address" :"false"
}
```


Field Description

Parameter Name	Example values	Parameter Type	Parameter Description
error_code	0	String	error_code
error_message	Success	String	error_message
result		Object	result
result.address_id	h5witQJeo6IQwz1qU6DNufUrBGmGW21u	String	result.address_id
result.document_detail		Object	result.document_detail
result.document_detail.name	**** profile	String	result.document_detail.name
result.document_detail.type	json	String	result.document_detail.type
result.document_detail.size	7300	Number	result.document_detail.size
result.document_temp_url_address	http://domain/open/ipfs/v1/tempUrl?id=iHbaqgLnZrGxFlTvBE2g7XvAWieWFYZM&expiredTi	String	result.document_temp_url_address

	me=7200&accessToken=OfepC2Atn9ZWEgU2bqbJ6rFqDtTrGv2V		

Example response


```
{
    "error_code" :"0",
    "error_message" :"Success",
    "result" {
        "address_id" :"h5witQJeo6IQwz1qU6DNufUrBGmGW21u",
        "document_detail" :{
            "name" :"**** profile",
            "type" :"json",
            "size" :7300
        },
"document_temp_url_address" :"http://domain/open/ipfs/v1/tempUrl?id=iHbaqgLnZrGxFlTvBE2g7XvAWieWFYZM&expiredTime=7200&accessToken=OfepC2Atn9ZWEgU2bqbJ6rFqDtTrGv2V",
    }
}
```


3.1.2 Storage files

Interface Status: Completed

Interface URL: http://domain/open/ipfs/v1/insertDocument

Content-Type: application/json

Request Method: post

Header parameters and description

Parameter Name	Example values	Parameter Type	Required or not	Parameter Description
accessToken	yhd3c9WqNwSKtP3XRQuJZm11OMxtYahN	String	Yes	

expiredTime	7200	Number	Yes	

Field Description

Parameter Name	Example values	Parameter Type	Required or not	Parameter Description
ipfs_id	EvqKdgbm0esJ1sMB	String	Yes	ipfs_id
address_id	itvqsYwXfNqWNfmrPe0I2x5HrOJuv6e7	String	Yes	address_id
space_id	F7Uo3dbKyUUp8f3EPU45hNvVsTFQwKjC	String	Yes	space_id

Body parameters and description


```
{
    "ipfs_id" :"EvqKdgbm0esJ1sMB",
    "address_id" :"itvqsYwXfNqWNfmrPe0I2x5HrOJuv6e7",
    "space_id" :"F7Uo3dbKyUUp8f3EPU45hNvVsTFQwKjC",
}
```


Field Description

Parameter Name	Example values	Parameter Type	Parameter Description
error_code	0	String	error_code
error_message	Success	String	error_message
address_id	itvqsYwXfNqWNfmrPe0I2x5HrOJuv6e7	String	address_id
result		Object	result
result.address_id	h5witQJeo6IQwz1qU6DNufUrBGmGW21u	String	result.address_id
result.storage_detail		Object	
result.storage_detail.name	Storage files	String	result.document_detail.name
result.storage_detail.type	json	String	result.document_detail.type
result.storage_detail.	7300	Number	result.document_det

size			ail.size
result.Storage_temp_url_address	http://domain/open/ipfs/v1/tempUrl?id=iHbaqgLnZrGxFlTvBE2g7XvAWieWFYZM&expiredTime=7200&accessToken=yhd3c9WqNwSKtP3XRQuJZm11OMxtYahN	String	result.Storage_temp_url_address

Example response


```
{
    "error_code" :"0",
    "error_message" :"Success",
    "address_id" :"itvqsYwXfNqWNfmrPe0I2x5HrOJuv6e7",
    "result" : {
        "address_id" :"h5witQJeo6IQwz1qU6DNufUrBGmGW21u",
        "storage_detail" :{
            "name" :"Storage files",
            "type" :"json",
            "size" :7300
        },
"Storage_temp_url_address" :"http://domain/open/ipfs/v1/tempUrl?id=iHbaqgLnZrGxFlTvBE2g7XvAWieWFYZM&expiredTime=7200&accessToken=yhd3c9WqNwSKtP3XRQuJZm11OMxtYahN",
    }
}
```


3.1.3 Update files

Interface Status: Completed

Interface URL：http://domain/open/ipfs/v1/updateDocument

Content-Type：application/json

Request Method：post

Header parameters and description

Parameter Name	Example values	Parameter Type	Required or not	Parameter Description
accessToken	LdIb1VA3iazOfT2E2HiGoKs9NiOrR2Y8	String	Yes	
expiredTime	7200	Number	Yes	

Field Description

Parameter Name	Example values	Parameter Type	Required or not	Parameter Description
ipfs_id	EvqKdgbm0esJ1sMB	String	Yes	ipfs_id
address_id	EzZgzRcHVzHizkiaEDkJEp7PHiPrIZ6j	String	Yes	address_id
space_id	eTEVraiauxJwafqRaZnBxXvn75gYoHYD	String	Yes	space_id
update_type	detail	String	Yes	update_type

Body parameters and description

```
{
    "ipfs_id" :"EvqKdgbm0esJ1sMB",
    "address_id" :"EzZgzRcHVzHizkiaEDkJEp7PHiPrIZ6j",
    "space_id" :"eTEVraiauxJwafqRaZnBxXvn75gYoHYD",
    "update_type" :"detail",
}
```

Field Description

Parameter Name	Example values	Parameter Type	Parameter Description
error_code	0	String	error_code
error_message	Success	String	error_message
result		Object	result
result.address_id	EzZgzRcHVzHizkiaEDkJEp7PHiPrIZ6j	String	result.address_id
result.update_detail		Object	result.update_detail
result.update_detail.name	Update files	String	result.update_detail.name

result.update_detail.type	json	String	result.update_detail.type
result.update_detail.size	7300	Number	result.update_detail.size
result.update_temp_url_address	http://domain/open/ipfs/v1/tempUrl?id=5sRcFIbJRa58HbJK2IcuZaLecJuLPfU4&expiredTime=7200&accessToken=LdIb1VA3iazOfT2E2HiGoKs9NiOrR2Y8	String	result.update_temp_url_address

Example response


```
{
    "error_code" :"0",
    "error_message" :"Success",
    "result" :{
        "address_id" :"EzZgzRcHVzHizkiaEDkJEp7PHiPrIZ6j",
        "update_detail" :{
            "name" :"Update files",
            "type" :"json",
            "size" :7300
        },

"update_temp_url_address" :"http://domain/open/ipfs/v1/tempUrl?id=5sRcFIbJRa58HbJK2IcuZaLecJuLPfU4&expiredTime=7200&accessToken=LdIb1VA3iazOfT2E2HiGoKs9NiOrR2Y8",
    }
}
```


3.1.4 Get information about users on the blockchain

Interface Status: Completed

Interface URL：http://domain/open/bigchaindb/v1/getIndividualDetail

Content-Type：application/json

Request Method：get

Header parameters and description

Parameter Name	Example values	Parameter Type	Required or not	Parameter Description
accessToken	DPGNfH865I2wGDxmZcEypyHnsJKZm0bU	String	Yes	
expiredTime	7200	Number	Yes	

Field Description

Parameter Name	Example values	Parameter Type	Required or not	Parameter Description
ipfs_id	EvqKdgbm0esJ1sMB	String	Yes	ipfs_id
address_id	EzZgzRcHVzHizkiaEDkJEp7PHiPrIZ6j	String	Yes	address_id
space_id	eTEVraiauxJwafqRaZnBxXvn75gYoHYD	String	Yes	space_id
update_type	detail	String	Yes	update_type

Body parameters and description


```
{
    "bigchaindb_id" :"LvHhnB8TxfE1IRJQ",
    "space_id" :"cmkIYIShvdkbGHNgFXrRADLndMYNtSpo",
}
```


Field Description

Parameter Name	Example values	Parameter Type	Parameter Description
error_code	0	String	error_code
error_message	Success	String	error_message
result		Object	result
result.address_id	EzZgzRcHVzHizkiaEDkJEp7PHiPrIZ6j	String	result.address_id
result.update_detail		Object	result.update_detail
result.update_detail.name	Update files	String	result.update_detail.name
result.update_detail.t	json	String	result.update_detail.t

ype			ype
result.update_detail.size	7300	Number	result.update_detail.size
result.update_temp_url_address	http://domain/open/ipfs/v1/tempUrl?id=5sRcFIbJRa58HbJK2IcuZaLecJuLPfU4&expiredTime=7200&accessToken=LdIb1VA3iazOfT2E2HiGoKs9NiOrR2Y8	String	result.update_temp_url_address

Example response


```
{
    "error_code" :"0",
    "error_message" :"Success",
    "result" :{
        "bigchaindb_id" :"LvHhnB8TxfE1IRJQ",
        "user_information" :{
            "name" :"***",
            "school" :"****"
            "account_number" :"********",
            "captchaOnOff" :"true",
            "uuid" :"m0o6qivkt9yscmu2ycphvn3s65y8pfyh"
        },
    }
}
```


3.1.5 Query Node Status

Interface Status: Completed

Interface URL：http://domain/open/bigchaindb/v1/getPeerStatus

Content-Type：application/json

Request Method：get

Header parameters and description

**Parameter Name**	**Example values**	**Parameter Type**	**Required or not**	**Parameter Description**
accessToken	UKhc57gSx82VQecz	String	Yes	

	h5NGTjpwCzOcYwoj			
expiredTime	7200	Number	Yes	

Field Description

Parameter Name	Example values	Parameter Type	Required or not	Parameter Description
peer_id	Xgfgc8HfDp7rMKHU	String	Yes	peer_id

Body parameters and description


```
{
    "peer_id" :"Xgfgc8HfDp7rMKHU",
}
```


Field Description

Parameter Name	Example values	Parameter Type	Parameter Description
error_code	0	String	error_code
error_message	Success	String	error_message
result		Object	result
result.address_id	EzZgzRcHVzHizkiaEDkJEp7PHiPrIZ6j	String	result.address_id
result.update_detail		Object	result.update_detail
result.update_detail.name	Update files	String	result.update_detail.name
result.update_detail.type	json	String	result.update_detail.type
result.update_detail.size	7300	Number	result.update_detail.size
result.update_temp_url_address	http://domain/open/ipfs/v1/tempUrl?id=5sRcFIbJRa58HbJK2IcuZaLecJuLPfU4&expiredTime=7200&accessToken=LdIb1VA3iazOfT2E2HiGoKs9NiOrR2Y8	String	result.update_temp_url_address

Example response

```
{
    "error_code" :"0",
    "error_message" :"Success",
    "result" :{
        "request_id" :"EzZgzRcHVzHizkiaEDkJEp7PHiPrIZ6j",
        "update_detail" :{
            "node_name" :"Update files",
            "node_state" :"Normal",
        },
    }
}
```

3.1.6 *Register Account*

Interface Status: Completed

Interface URL：*http://domain/open/bigchaindb/v1/registerIndividualAccount*

Content-Type：*application/json*

Request Method：*post*

Header parameters and description

Parameter Name	Example values	Parameter Type	Required or not	Parameter Description
accessToken	YysRrnssVrq0u1U0qORCPSPZVxICqltc	String	Yes	
expiredTime	7200	Number	Yes	

Field Description

Parameter Name	Example values	Parameter Type	Required or not	Parameter Description
name	***	String	Yes	result.document_detail.name
password	cWusTDiC	String	Yes	password

Body parameters and description

```
{
    "name": "***",
    "password": "cWusTDiC"
}
```

Field Description

Parameter Name	Example values	Parameter Type	Parameter Description
error_code	0	String	error_code
error_message	Success	String	error_message
user		Object	user
user.gender	male	String	user.gender
user.avatar	/uploads/default_avatar.png	String	user.avatar
user.following	{}	Object	user.following
user._id	5f7c5127354939e9ce5465a2	String	user._id
user.name	***	String	result.document_detail.name
user.password	cWusTDiC	String	user.password
user.createdAt	2022-7-06T11:12:39.664Z	String	user.createdAt
user.updatedAt	2022-7-06T11:12:39.664Z	String	user.updatedAt
user.__v	0	Number	

Example response

```
{
    "error_code" :"0",
    "error_message" :"Success",
    "user": {
        "gender": "male",
        "avatar": "/uploads/default_avatar.png",
        "following": [],
        "_id": "5f7c5127354939e9ce5465a2",
        "name": "***",
```

```
            "password": "cWusTDiC",
            "createdAt": "2022-7-06T11:12:39.664Z",
            "updatedAt": "2022-7-06T11:12:39.664Z",
            "__v": 0
      }
}
```


Field Description

Parameter Name	Example values	Parameter Type	Parameter Description
status	400001	Number	status
message	User name already exists, please change a name	String	message

Example response


```
{
      "status": 400001,
      "message": "User name already exists, please change a name"
}
```


3.1.7 Configure blockchain property files

Interface Status: Completed

Interface URL：http://domain/open/bigchaindb/v1/configChainProperties

Content-Type：application/json

Request Method：post

Header parameters and description

Parameter Name	Example values	Parameter Type	Required or not	Parameter Description
accessToken	2EGz4EpthaqTzY8XoaiRQJmVSo0HQ8lX	String	Yes	
expiredTime	7200	Number	Yes	

Field Description

Parameter Name	Example values	Parameter Type	Required or not	Parameter Description
accessToken	2EGz4EpthaqTzY8XoaiRQJmVSo0HQ8lX	String	Yes	accessToken
number_of_nodes	**	String	Yes	number_of_nodes
node_Relationships	sibling	String	Yes	node_Relationships
node_Name	***	String	Yes	node_Name
space_id	PnUc1ccX4axDBM9WrRgCXtdMdgFM9wrz	String	Yes	space_id

Body parameters and description

```
{
    "accessToken" :"2EGz4EpthaqTzY8XoaiRQJmVSo0HQ8lX",
    "number_of_nodes" :"**",
    "node_Relationships" :"sibling",
    "node_Name" :"***" ,
    "space_id" :"PnUc1ccX4axDBM9WrRgCXtdMdgFM9wrz",
}
```

Field Description

Parameter Name	Example values	Parameter Type	Parameter Description
error_code	0	String	error_code
error_message	Success	String	error_message
space_id	PnUc1ccX4axDBM9WrRgCXtdMdgFM9wrz	String	space_id
result		Object	result
result.node_Name	***	String	result.node_Name
result.updatedAt	2022-7-06T11:12:39.664Z	String	result.updatedAt

Example response

```
{
```

```
    "error_code" :"0",
    "error_message" :"Success",
    "space_id" :"PnUc1ccX4axDBM9WrRgCXtdMdgFM9wrz",
    "result": {
        "node_Name" :"***" ,
        "updatedAt": "2022-7-06T11:16:32.642Z",
    }
}
```
